# Randomized, Placebo‐Controlled, First‐in‐Human Study of the Safety, Tolerability, and Pharmacokinetics of Single and Repeat Oral Doses of Novel Antitubercular Drug Candidate, GSK2556286, in Healthy Adults

**DOI:** 10.1002/jcph.70203

**Published:** 2026-05-15

**Authors:** Emma Ilsley, Edward Banham‐Hall, Tetyana Chaychenko, Katerina Chinenyeze, Gareth Maher‐Edwards, Catherine Muya, Katie Rolfe, Raman Sharma, Simon Tiberi, David Barros‐Aguirre

**Affiliations:** ^1^ GSK London UK; ^2^ GSK Cambridge UK; ^3^ GSK Stevenage UK; ^4^ Blizard Institute Barts and The London School of Medicine and Dentistry Queen Mary University of London London UK; ^5^ GSK Tres Cantos Spain

**Keywords:** dose escalation, first‐in‐human, pharmacokinetics, safety, tuberculosis, GSK2556286

## Abstract

This first‐in‐human study (ClinicalTrials.gov identifier: NCT04472897) evaluated the safety, tolerability, pharmacokinetics, and food effect of single ascending and repeat oral doses of GSK2556286 (GSK286), a novel antitubercular drug candidate with efficacy in animal models of tuberculosis. In part A, 56 participants received single GSK286 doses (25–1000 mg; 10 cohorts) and 19 participants received a placebo. In part B, GSK286 and placebo doses were administered once daily for up to 14 days. Cohorts received repeat doses of GSK286 (225 or 650 mg; 13 participants) or placebo (four participants) in part B before the study was placed on hold due to reaching the stopping criterion, defined as clinically significant, non‐serious adverse events (AEs) that are considered to be at least possibly related to GSK286 in two or more participants on active treatment. Single GSK286 doses were well tolerated; all reported AEs were mild, apart from moderate AEs in two participants administered GSK286 1000 mg, and there were no serious AEs (SAEs). In part B (repeat dosing), three participants reported moderate AEs, one reported a severe AE, and there were no SAEs. However, four of six participants in the 650 mg repeat dose cohort discontinued treatment due to the AEs (at least one) of headache, photophobia, fatigue, nausea, and vomiting. There was high intraindividual variability in the pharmacokinetics of GSK286 in both parts of the study, and the effect of food in modulating this variability was unclear. The combination of unexpected pharmacokinetic variability and exposure‐related AEs led to termination of the study.

## Introduction

Tuberculosis (TB), caused by *Mycobacterium tuberculosis*, was the world's leading cause of death from a single infectious agent in 2024, with 1.23 million estimated deaths, surpassing the number of deaths caused by both COVID‐19 and HIV/AIDS.^1^ In recent decades, different antitubercular drugs and treatment regimens have been introduced, but with challenges associated with toxicity, poor outcomes, and lengthy treatment duration (at least 6 months) for most patients.^2^, ^3^ Also, drug resistance is a health security threat; in 2024, around 400,000 people developed multidrug‐resistant or rifampicin‐resistant TB.^1^

New safe and effective treatments, with novel mechanisms of action and short‐course regimens, are needed to meet the United Nation's global pledge to end the TB epidemic.^1^, ^4^ Compounds with selective activity within macrophages and cholesterol‐dependent activity against *M*. *tuberculosis* were identified as a promising area of research.^5^ This led to the development of GSK2556286 (GSK286), a pyrimidine derivative that interferes with mycobacterial cholesterol catabolism and inhibits *M*. *tuberculosis* extracellularly in the presence of cholesterol and within human macrophages.^6^, ^7^ Preliminary in vitro data (recombinant human enzymes) indicate oxidative metabolism of GSK286, predominantly via CYP3A4. In humans, metabolites in plasma and urine form via oxidation, N‐ and O‐dealkylation, and sulfation, while in vivo evidence (nonclinical and clinical) of metabolic bioactivation is consistent with acyl glucuronide formation and glutathione conjugation.^8^, ^9^

GSK286 had favorable in vivo efficacy and safety profiles in animal models and showed the potential to shorten TB treatment.^7^, ^10^, ^11^ GSK286 also had an additive effect to a bedaquiline‐pretomanid (BPa) combination therapy regimen in a BALB/c mouse model of TB, suggesting the potential to replace linezolid (L) in a BPaL regimen.^7^

This phase 1, first‐in‐human (FIH) study evaluated the safety, tolerability, and pharmacokinetics of single and repeat ascending oral doses of GSK286, and the food effect of an oral dose, to establish safety cover and characterize its pharmacokinetics for further studies. The study was terminated because of a combination of unexpected pharmacokinetic variability and the observation of exposure‐related adverse events (AEs), which reduced the predicted therapeutic window. Here, we describe the safety and pharmacokinetic profiles of GSK286 in this study of healthy adults.

## Methods

### Ethics

The study protocol and study documents were reviewed and approved by independent ethics committees: Stichting Beoordeling Ethiek Biomedisch Onderzoek in the Netherlands and Health and Social Care Research Ethics Committee A in the UK. All participants provided written informed consent. The study was conducted between October 2020 and November 2024 at one center in the Netherlands (QPS Netherlands) and one in the UK (Clinical Unit Cambridge), according to the Declaration of Helsinki and Good Clinical Practice guidelines. The first three cohorts in study part A were conducted in the Netherlands; thereafter, the study was conducted in the UK only.

### Study Design and Participants

This was a randomized, placebo‐controlled FIH study (ClinicalTrials.gov identifier, NCT04472897) that was double‐blinded with respect to the allocation of GSK286 or placebo to participants. Selected sponsor staff were unblinded after the study was put on hold.

Enrolled participants were healthy, aged 18–60 years, had body weight ≥50 kg and body mass index 19–29.9 kg/m^2^, and if aged 51–60 years, had received at least one dose of a COVID‐19 vaccine at least 3 weeks before signing the consent form. Full inclusion and exclusion criteria are provided in the study protocol, which is available at https://www.gsk‐studyregister.com/trials/210035.

The primary study objectives were to investigate the safety and tolerability of GSK286 after single ascending and repeat oral doses, and to determine the pharmacokinetics of GSK286 in healthy adult participants. Secondary study objectives were to assess the effect of food on the pharmacokinetics of GSK286 following an oral dose, the preliminary dose proportionality of GSK286 following single and repeat oral doses, and to examine the extent of accumulation and achievement of steady state following repeat oral doses of GSK286.

Cohorts of eight participants each were randomized to GSK286 or placebo (both tablet formulations) in a 6:2 ratio. Each participant was randomized to one cohort only. As this was an FIH study, dosing for each cohort began with a sentinel group of two participants (one received GSK286, one placebo).

The study had two parts: part A had a single ascending dose, sequential, parallel design, and part B had a multiple ascending dose, sequential, parallel design that tested repeated doses. Progression from part A to part B was based on acceptable safety, tolerability, and pharmacokinetic profiles in part A. In both parts, all screening assessments were completed within 28 days before dose administration, and follow‐up was to be 7 to 14 days after the last GSK286 administration.

In part A, each participant received a single oral dose of either GSK286 or a matching placebo on day 1. The planned starting dose of GSK286 was 25 mg (cohort 1A), with maximum exposure determined by the predicted targeted therapeutic exposure translated from a preclinical study of GSK286 that used a mouse model of TB (GSK, data on file). Participants were discharged on day 4, after completion of all assessments. Dose escalation took place following a data review for each cohort by the Dose Escalation Committee.

Following initial dosing of cohorts in the fasted state, the effect of food (high‐fat meal) on the safety, tolerability, and pharmacokinetics of a single GSK286 dose was first investigated in cohort 4A (75 mg), in parallel with the ascending dose cohorts. Subsequent cohorts (5A to 10A) were to receive doses in the fed (high‐fat meal) or fasted state, as determined by the Dose Escalation Committee based on emerging data.

In part B, each participant was randomized to receive a daily dose of GSK286 or a matching placebo over a period of up to 14 days, and it was planned that participants would remain in the unit until day 17. The first repeat dose cohort (1B, 225 mg daily) included an assessment of food effect, with participants administered doses after a standard‐fat meal on all days apart from day 9, when they were administered the dose when fasted, and day 11, when they were administered the dose after a high‐fat meal. Appropriate doses and dose regimens for part B were selected by the Dose Escalation Committee based on results from part A and any preceding repeat dose cohorts from part B.

Participants were not permitted to take any prescription or non‐prescription drug within 7 days before the start of the study until completion of follow‐up. Paracetamol administration (dose ≤2 g/day) was permitted during the study.

### Safety Assessments

The primary safety endpoints were the number and intensity of serious and non‐serious AEs. Each AE and serious AE (SAE) reported during the study was assessed by study investigators for its intensity, using the Division of AIDS (DAIDS) grading scale,^12^ and causal relationship to the study drug.

Data on vital signs (blood pressure, heart rate, temperature, and respiratory rate) and electrocardiogram (ECG) data (via 12‐lead ECGs and continuous 24 h Holter monitoring) were recorded at pre‐defined timepoints. Telemetry was recorded from at least 15 min pre‐dose to 24 h post‐dose.

Urine safety assessments were conducted at screening and on days 1 and 2 in part A, and on days 1, 4, 8, and 14 in part B. Clinical hematology and chemistry analyses were conducted pre‐dose and on days 2 and 4, and at follow‐up in part A, and on days 3, 6, 10, 13, and 16, and at follow‐up in part B.

### Pharmacokinetic Assessment

Blood samples for pharmacokinetic analysis were collected pre‐dose and 15, 30, and 45 min, and 1, 1.5, 2, 3, 4, 6, 8, 12, 15, 24, 36, 48, and 72 h after the single dose in part A and, in part B, at the same time intervals after the first dose administration on day 1 and last dose administration on day 14. Samples were also taken pre‐dose on days 6, 7, and 8 in part B. For the food effect cohort in part B, blood samples were also to be collected pre‐dose and 15, 30, and 45 min, and 1, 1.5, 2, 3, 4, 6, 8, 12, 15, and 24 h post‐dose on days 9 and 11.

GSK286 plasma concentrations were quantified using an in‐house assay that was validated for concentrations 2–2000 ng/mL. This assay showed precision through within‐run and between‐run coefficient of variation calculations of ≤7.72% and ≤10.04%, respectively, and accuracy through bias calculations of −9.50% ≤ bias ≤ 9.33% (within‐run accuracy) and −3.50% ≤ bias ≤ −1.25% (average between‐run accuracy).

Primary pharmacokinetic endpoints for part A included area under the plasma drug concentration versus time curve (AUC) from time 0 to time of last quantifiable concentration (AUC_0‐t_) and to infinity (AUC_0‐∞_), maximum observed plasma drug concentration (C_max_), time to maximum observed plasma drug concentration (T_max_), and terminal phase half‐life (T_1/2_). For part B, primary pharmacokinetics endpoints were AUC_0–t_, AUC_0–∞_, AUC from time 0 to end of dosing period (AUC_0–τ_), C_max_, T_max_, trough plasma concentration (C_τ_), and T_1/2_. The same pharmacokinetic parameters were calculated in the assessment of food effect as a secondary objective following single dose administration (in fasted and fed conditions) in part A and repeated dose administration (in fasted, fed with normal meal, and fed with high‐fat meal conditions) in part B to exclude the effect of between‐subject variability.

The dose proportionality assessment was to be conducted using AUC_0‐t_, AUC_0‐∞_, and C_max_ for part A, and AUC_0‐τ_ and C_max_ for part B. The observed accumulation ratio was based on AUC and C_max_ and the steady‐state ratio following repeat dosing (day 14 versus day 1). Trough plasma concentrations at the end of the dosing interval (Cτ) were to be calculated to assess if GSK286 steady state was achieved following repeat oral doses.

### Statistical Analysis

No formal statistical hypotheses were tested. The safety and tolerability primary endpoints were evaluated by summarizing the number and severity of serious and non‐serious AEs using descriptive statistics. Pharmacokinetic parameters for each participant were estimated by standard noncompartmental analysis using WinNonlin versions 8.1 to 8.3, based on actual sampling times. Geometric mean values, their 95% confidence intervals (CIs), and between‐patient coefficients of variation (% CVb) were calculated for AUC, C_max_, and C_τ_. Median and minimum/maximum T_1/2_ and T_max_ values were calculated.

In the analysis of dose proportionality, a fixed power model was used, analyzing log‐transformed plasma GSK286 pharmacokinetic parameters (AUC and C_max_). Log(dose) was included as a fixed effect, and the mean slope and its 90% CI calculated. A fixed effects model including log(dose), day, and log(dose) × day was to be used to assess accumulation. For each dose, the day 14: day 1 ratio and 90% CI of plasma GSK286 pharmacokinetic parameters were to be calculated. If log(dose) and the log(dose) × day interaction terms were not statistically significant, then a single ratio (90% CI) pooled across all doses was to be calculated.

Achievement of steady state was assessed by analyzing C_τ_. A fixed effects model including log(dose), day, and log(dose) × day was to be used, and the slope for the day effect for each dose, with 90% CI, was examined to determine steady state. If the log(dose) × day interaction term was not statistically significant, then a single slope (90% CI) pooled across all doses was to be calculated.

For the analysis of food effect on the pharmacokinetic parameters on AUC, C_max_, T_1/2_, and C_τ_, fixed effects models were fitted for part A, with fed/fasted state, dose, and fed/fasted state × dose fitted as covariates. Only data from the UK site were included in the analysis (cohorts 4A to 10A). For part B, mixed effects models were fitted with food state (high‐fat meal/standard meal/fasted) fitted as a fixed effect and participant as a random effect. Point estimates and corresponding 90% CIs were estimated from the models for the geometric mean ratios of fed:fasted (derived separately for each dose in part A, and for high‐fat meal:fasted and standard meal:fasted in part B). For T_max_, median differences between fed and fasted cohorts (with 90% CIs calculated using the Lehmann estimation) were calculated.

No formal interim analyses were planned. Safety, tolerability, and pharmacokinetics data were reviewed before each dose escalation in part A (single dose) and part B (repeat dose), and before and after investigation of the food effect.

All analyses were performed using SASviya4 software.

## Results

### Participants and Cohorts

Of 256 screened participants, 92 were eligible and enrolled in study parts A (75 participants) and B (17 participants). All participants were male, with a mean age (standard deviation) of 36.1 (10.9) years in part A and 37.2 (9.4) years in part B, and most participants (64 participants, 85%, in part A; 14 participants, 82%, in part B) were White. Mean body mass index (standard deviation) was 24.9 (2.7) kg/m^2^ in part A and 24.5 (2.3) kg/m^2^ in part B.

In part A, 19 participants received placebo, 27 participants (cohorts 1A, 2A, 3A, 4A, and 6A) received a single dose of GSK286 (respectively, 25, 75, 225, 75, and 225 mg) under fasting condition, and 29 participants (cohorts 5A, 7A, 8A, 9A, and 10A) received a single dose of GSK286 (respectively, 75, 225, 450, 650, and 1000 mg) under fed condition (high‐fat meal). All 75 participants completed study part A. Seven (9%) participants had protocol deviations, most commonly related to study procedures and biological sample specimen procedures (four and three participants, respectively).

In part B, four participants were enrolled to receive a placebo, seven to receive GSK286 225 mg repeat dose (1B cohort), and six to receive GSK286 650 mg repeat dose (2B cohort). Six (86%) participants in the 1B cohort completed the study, and one (14%) participant withdrew due to an AE.

The study was placed on temporary hold on March 5, 2024, due to reaching the stopping criterion, defined in the protocol as clinically significant, non‐serious AEs that are considered to be at least possibly related to GSK286 in two or more participants on active treatment, in the 2B cohort. All participants in the 2B cohort were withdrawn, either due to an AE (four of six) or the physician's decision (two of six). One of these six participants completed 14 days of treatment before withdrawal due to the physician's decision. None of the AEs were categorized as serious, and all symptoms fully resolved following withdrawal of GSK286.

### Safety and Tolerability

At least one AE was reported by 22 of 75 (29%) participants in part A and 14 of 17 (82%) participants in part B. In both parts of the study, most reported AEs had mild (grade 1) severity (Tables 1 and 2). Three participants reported moderate (grade 2) AEs in part A; one in the placebo group and two in the cohort that received GSK286 1000 mg (Table 1). In part B, three participants reported moderate AEs (one received GSK286 225 mg, two received GSK286 650 mg), and one participant who received GSK286 650 mg reported a severe (grade 3) AE (Table 2).

**Table 1:** Overview of Adverse Events in Part A (Single Dose)

	Number (percentage) of participants reporting adverse events										
AE	Placebo (N = 19)	GSK286, 25 mg (cohort 1A, N = 6)	GSK286, 75 mg (cohort 2A, N = 6)	GSK286, 75 mg (cohort 4A, N = 6)	GSK286, 75 mg, fed (cohort 5A, N = 6)	GSK286, 225 mg (cohort 3A, N = 3)	GSK286, 225 mg (cohort 6A, N = 6)	GSK286, 225 mg, fed (cohort 7A, N = 6)	GSK286, 450 mg, fed (cohort 8A, N = 6)	GSK286, 650 mg, fed (cohort 9A, N = 6)	GSK286, 1000 mg, fed (cohort 10A, N = 5)
Any	6 (32)	1 (17)	1 (17)	1 (17)	0	1 (33)	3 (50)	1 (17)	2 (33)	3 (50)	3 (60)
Mild	5 (26)	1 (17)	1 (17)	1 (17)	0	1 (33)	3 (50)	1 (17)	2 (33)	3 (50)	1 (20)
Moderate	1 (5)	0	0	0	0	0	0	0	0	0	2 (40)
Severe	0	0	0	0	0	0	0	0	0	0	0
Exposure‐related	0	1 (17)	1 (17)	0	0	1 (33)	0	0	0	0	2 (40)
Leading to discontinuation	0	0	0	0	0	0	0	0	0	0	0
SAE	0	0	0	0	0	0	0	0	0	0	0

AE, adverse event; GSK286, GSK2556286; N, number of participants in group; SAE, serious adverse event.

**Table 2:** Overview of Adverse Events in Part B (Repeat Dose)

	Number (percentage) of participants reporting adverse events		
AE	Placebo (N = 4)	GSK286, 225 mg (cohort 1B, N = 7)	GSK286, 650 mg (cohort 2B, N = 6)
Any	3 (75)	5 (71)	6 (100)
Mild	3 (75)	4 (57)	3 (50)
Moderate	0	1 (14)	2 (33)
Severe	0	0	1 (17)
Exposure‐related	1 (25)	2 (29)	4 (67)
Leading to discontinuation	0	1 (14)	4 (67)
SAE	0	0	0

AE, adverse event; GSK286, GSK2556286; N, number of participants in group; SAE, serious adverse event.

Five participants in part A, all of whom had received GSK286, reported exposure‐related AEs (urinary sediment present in one participant in cohort 1A; headache in one participant in cohort 2A; abdominal pain and fatigue in one participant in cohort 3A; postural dizziness, abdominal pain, diarrhea, nausea, vomiting, loss of appetite, and headache in one participant in cohort 10A; headache and decreased appetite in one participant in cohort 10A). In part B, seven participants reported exposure‐related AEs, six of whom had received GSK286 (in cohort 1B, diarrhea and abdominal pain in one participant, and decreased appetite in one participant; in cohort 2B, fatigue, photophobia, headache, nausea, and vomiting in one participant, headache in two participants, and headache and photophobia in one participant).

There were no SAEs and no deaths reported in the study, and there were no clinically significant changes from baseline in clinical laboratory values, ECG findings, or vital signs (data not shown).

In both parts of the study, the most frequently reported AE was headache (Table 3), which was reported by eight (11%) participants in part A and six (35%) in part B. Headache severity was mild for six participants and moderate for two participants in part A, and, in part B, mild for three participants, moderate for two participants, and severe for one participant. Two participants in part A and two in part B reported headache with associated symptoms of nausea, vomiting, blurred vision, and/or photophobia, while the remaining reports (six participants in part A, four in part B) were without associated symptoms. All reports of headache, with or without other associated symptoms, were non‐serious and resolved by the end of the day after GSK286 was discontinued, and with paracetamol treatment for some participants.

**Table 3:** Adverse Events by Preferred Term that were Reported by More than One Study Participant in Part A (Single Dose) and Part B (Repeated Dose)

	Number (percentage) of participants reporting AE	
Preferred term	Part A (N = 75)	Part B (N = 17)
Any	22 (29)	14 (82)
Headache	8 (11)	6 (35)
Abdominal pain	2 (3)	2 (12)
Nausea	2 (3)	2 (12)
Dermatitis contact	2 (3)	2 (12)
Diarrhea	1 (1)	3 (18)
Decreased appetite	1 (1)	2 (12)
Vomiting	1 (1)	2 (12)
Fatigue	1 (1)	2 (12)
Medical device reaction/dermatitis	1 (1)	2 (12)
Nasal congestion	0	2 (12)
Photophobia	0	2 (12)
Contusion	2 (3)	1 (6)
Pain in extremity	2 (3)	0
Dizziness	2 (3)	0

AE, adverse event; N, number of participants in group.

All AEs had mild (grade 1) intensity apart from, in part A, three participants reported moderate intensity (grade 2) events (two reports of headache; one report of pain in extremity); in part B, three participants reported moderate intensity events (two reports of headache; one report each of diarrhea, abdominal pain, catheter site cellulitis, and thrombophlebitis) and one participant reported severe (grade 3) headache.

None of the AEs in part A led to study discontinuation. Five (29%) participants in part B discontinued due to an AE. One participant in the 225 mg repeat dose cohort (1B) discontinued due to thrombophlebitis and other AEs (drug intolerance, pain, catheter site cellulitis, and nasal congestion) not related to study treatment. In the 650 mg repeat dose cohort (2B), four of the six participants discontinued study treatment due to the AEs (at least one) of headache, photophobia, fatigue, nausea, and vomiting.

### Pharmacokinetic Profile

**Figure 1:**
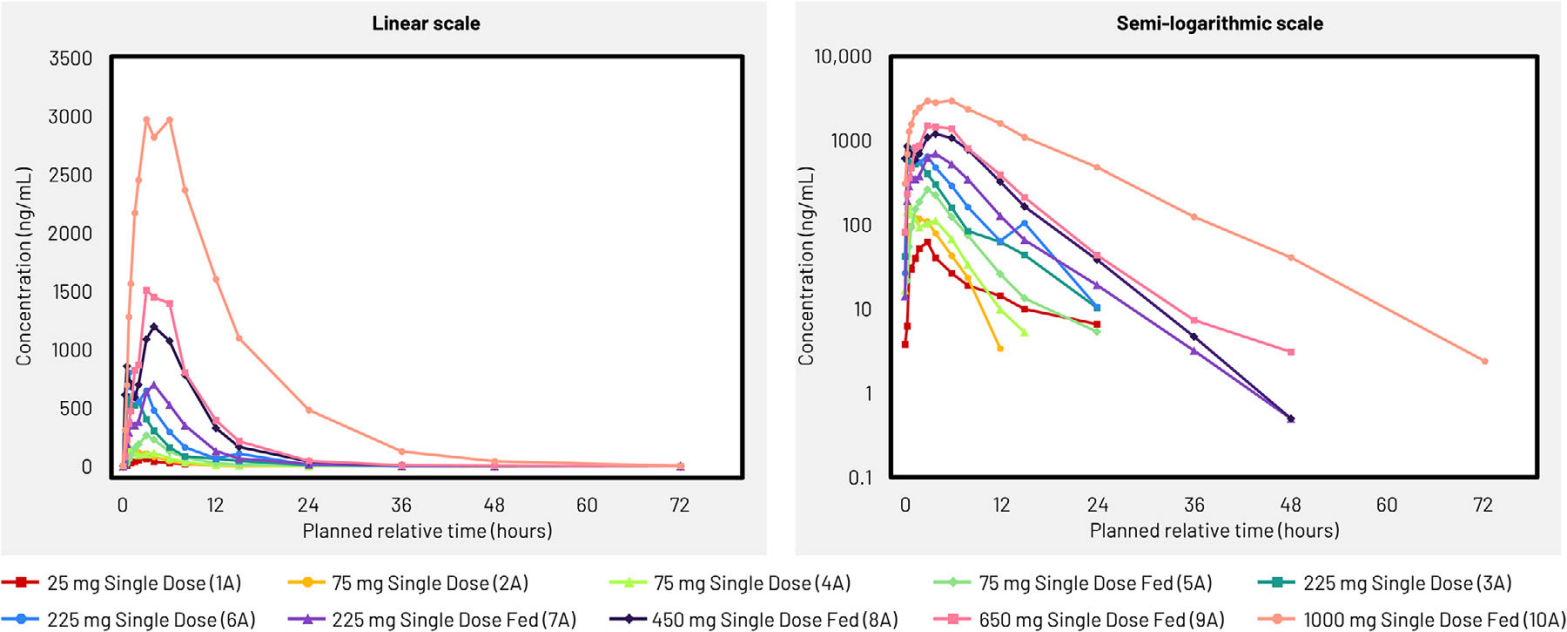
Mean plasma GSK2556286 concentration–time plots (linear and semi‐log) for part A (single dose). Lower limit of quantification, 20 ng/mL for cohorts 1A to 6A and 2 ng/mL for other cohorts.

**Figure 2:**
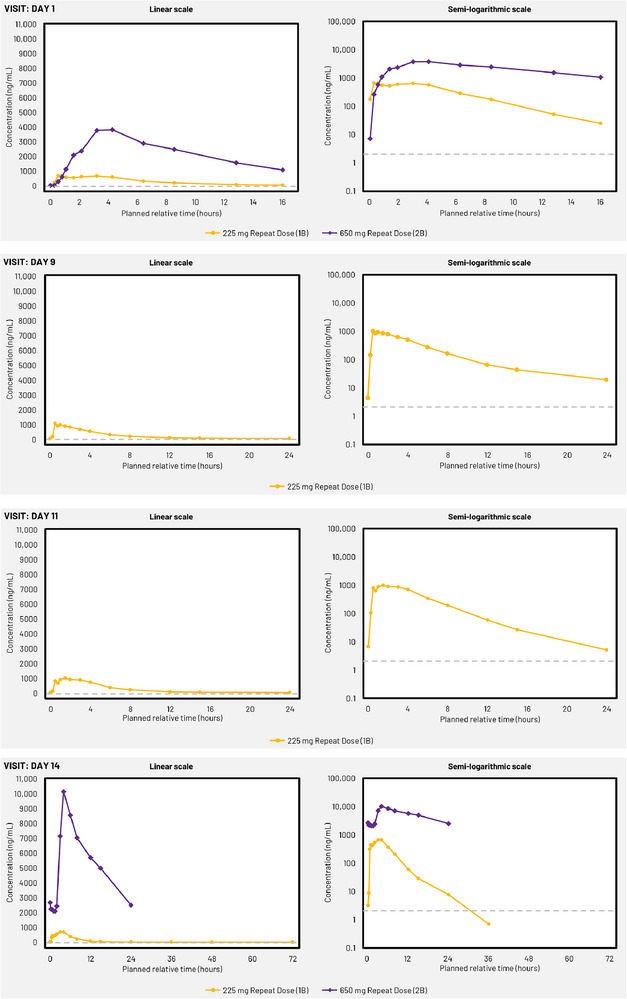
Mean plasma GSK2556286 concentration–time plots (linear and semi‐log) for part B (repeated doses) at days 1, 9, 11, and 14. Lower limit of quantification, 2 ng/mL (indicated by dashed lines). Records over 24 h post‐last dose excluded for participants who discontinued the study drug.

Mean plasma concentrations of GSK286 increased with dose, with concentrations peaking 1.5–4 h after GSK286 administration in both study parts (Figures 1 and 2).

In part A, across the 75–1000 mg dose range under fed conditions, intraindividual variability was high for AUC_0‐t_ and C_max_ (CV_b_, 48%–87% and 21%–63%, respectively) (Table S1). There was a greater than dose‐proportional increase in slope point estimates for AUC_0–t_ and AUC_0–∞_ following single doses, with slope estimates of 1.20 (90% CI: 0.988, 1.416) and 1.21 (90% CI: 0.992, 1.430), respectively. The slope estimate for C_max_ was slightly less than dose‐proportional, with a slope estimate of 0.95 (90% CI: 0.812, 1.078). This non‐proportionally was not considered significant because the 90% CI for all slopes included 1.0.

In part B, for both the 225 and 650 mg repeat doses, intraindividual variability was also high for AUC_0‐t_ and C_max_ (CV_b_ 32%–90% and 30%–67%, respectively) (Table S1). For the 650 mg repeat dose, geometric mean values for AUC_0‐t_, AUC_0‐∞_, and C_max_ at day 1 were significantly increased in comparison to values for the 225 mg repeat dose cohort (approximately seven to nine‐fold increase for exposures and four‐fold increase for C_max_, compared to three‐fold increased dose) (Table S1). Comparison of day 1 values in the 650 mg repeat dose cohort (part B) with day 1 values following the 650 mg single dose cohort (part A) showed three‐fold and two‐fold increases in geometric mean values for AUC_0‐t_ and C_max_, respectively (Table S1).

In the analysis of the food effect in part A, administration of GSK286 75 mg single dose under fed conditions resulted in increases in AUC_0‐t_, AUC_0‐∞_, and C_max_, with a delay in T_max_ versus the fasted state (Table S2). When GSK286 225 mg single dose was administered under fed conditions, both AUC_0‐t_ and AUC_0‐∞_ increased by less than 10%, while C_max_ was approximately 20% lower compared to fasted conditions (Table S2). All 90% CIs for ratios of fed:fasted condition for each parameter included 1.0, apart from AUC_0–t_ for 75 mg single dose (ratio 2.02; 90% CI: 1.05, 3.89), so the food effect was not considered to be clinically relevant.

In the analysis of the food effect in part B, administration of GSK286 225 mg with a high‐fat meal resulted in slight increases in point estimates of geometric mean AUC_0‐t_, AUC_0‐∞_, AUC_0‐τ_, and C_max_ compared with fasted conditions (Table S3). Administration of the same dose with a standard meal resulted in slight decreases in these parameters compared to fasted conditions (Table S3). In both conditions, the 90% CIs for ratios of point estimates of C_max_ and AUC parameters encompassed 1.0, indicating no significant differences compared with fasted conditions, although T_max_ was delayed (median 1.5 h with high‐fat meal and 3.0 h with standard meal versus 0.6 h in fasted condition) and C_τ_ was significantly decreased (3.5 and 4.9, respectively vs. 14.1).

Due to the inclusion of only one cohort (administered 225 mg dose) in part B, the log(dose) and log(dose) × day covariates were not included in the calculations of both accumulation and steady state. Assessment of the accumulation ratio, day 14 versus day 1, showed repeat daily dosing of GSK286 225 mg resulted in 1.14‐fold (90% CI: 0.70, 1.85) accumulation based on AUC_0–τ_, while there was no accumulation in C_max_ (accumulation ratio 0.92; 90% CI: 0.56, 1.50). Therefore, overall, there was no clear evidence for accumulation. No accumulation calculations were made following GSK286 650 mg repeat doses due to treatment withdrawal for most participants. However, pharmacokinetic parameters for the single participant who completed 14 days of repeat 650 mg doses had an approximately 3‐fold increase in AUC_0–τ_ (44,199 to 125,159 ng × h/mL) and 2‐fold increase in C_max_ (4960 to 10,100 ng/mL) from day 1 geometric mean values. Assessment of C_τ_ for the 225 mg repeat dose cohort from day 1 to day 14 indicated a back‐transformed slope value of 1.0374 (90% CI: 0.9737, 1.1054), which was indicative of the achievement of steady state.

## Discussion

GSK286 is a novel first‐in‐class antitubercular compound that was shown in preclinical studies to inhibit *M*. *tuberculosis* growth within human macrophages, with activity against extracellular bacteria in cholesterol‐containing culture medium, and no cross‐resistance with other antitubercular drugs.^7^, ^10^, ^11^ This pyrimidine derivative had properties in mouse models of TB similar to that seen with pyrazinamide, rifampicin, and bedaquiline, with the potential to enable more effective and shorter TB drug regimens with activity against drug‐susceptible and drug‐resistant TB.^6^, ^7^, ^10^, ^11^ Also, the preclinical safety profile supported the progression to clinical development.^7^

This FIH study evaluated the safety, tolerability, and pharmacokinetics profile of single and repeat oral doses and the food effect of GSK286 in healthy adult participants. The study had two parts. In part A, participants received GSK286 25–225 mg as a single dose under fasting conditions, GSK286 75–1000 mg as a single dose under fed conditions, or a matching placebo. Part B was terminated after the first two GSK286 cohorts, which received repeat doses of 225 mg (cohort 1B) or 650 mg (cohort 2B).

In study part A, GSK286 was generally well tolerated, and in both study parts, most reported AEs were mild, and there were no SAEs. There were no clinically significant changes from baseline clinical laboratory, ECG, or vital sign values. The most frequently reported AE in both study parts was headache, reported by 11% of participants in part A and 35% in part B, which was sometimes reported with associated symptoms of nausea, vomiting, blurred vision, and photophobia, and sometimes without other symptoms. All reports of headache were non‐serious. In part A, two participants reported grade 2 headache, and in part B, two participants reported grade 2 headache, and one reported grade 3 headache. All resolved by the end of the day after discontinuation of GSK286. No safety signals were observed in the nonclinical studies^7^ that were indicative of the headaches and other symptoms observed in the healthy volunteers.

Day 1 pharmacokinetic parameters were compared between the cohorts that received a single oral dose (cohorts 1A, 2A, etc.) and those that received repeat oral doses (cohorts 1B and 2B). This showed much higher exposure after a single 650 mg dose in cohort 2B as compared to cohort 9A (650 mg single dose) and cohort 1B (225 mg repeat dose), which could not be explained despite thorough investigations. This coincided with a high incidence of headache (with or without associated symptoms) among the six participants in the 650 mg repeat dose cohort. Four of these participants discontinued study treatment due to the AEs of (at least one) headache, photophobia, fatigue, nausea, and vomiting. This led to the study being temporarily halted because of the combination of unexpected pharmacokinetic variability (as discussed further below) and the exposure‐related AEs that were observed in the study. A post‐hoc analysis showed that AEs that were judged by the investigator to be exposure‐related were also reported in two participants in the 225 mg repeat dose cohort.

High intraindividual variability in the pharmacokinetics of GSK286 was observed in both parts of the study and food had an unclear effect in modulating this variability. The effect of the high‐fat fed condition was not clinically significant in part A or B, with point estimates of ratios trending in opposite directions in part B for high‐fat and standard‐fat fed conditions. Intraindividual variability among the cohorts possibly convoluted the observation in part A. Also, dose proportionality was only calculable in part A and, whilst there was a slightly greater than dose‐proportional increase in AUC based on the slope point estimates, this was not statistically significant (CIs for the slope included 1). Drug accumulation was not found to be clinically significant in the GSK286 225 mg repeat dose cohort, with pharmacokinetic parameters indicating that steady state was reached by day 14. There were insufficient data to formally assess accumulation in the 650 mg repeat dose cohort, although the participant who completed 14 days of repeat doses did show evidence of accumulation.

A thorough and detailed review of the human safety and pharmacokinetic data was conducted by GSK clinical, safety, and pharmacokinetics experts in an effort to identify the origin of the increased exposure in cohort 2B (650 mg repeat dose). Full drug accountability was conducted at site and the process for dispensing and administering doses was fully assessed to ensure that all participants had received the correct dose as per protocol. No anomalies were found in drug accountability, and the process was found to be extremely robust. Additional analyses were performed, including the comparison of clinical release test data for the two batches of 250 mg tablets used in this study. This showed no significant differences, and biorelevant dissolution testing and testing with a benchtop gastrointestinal model that simulates the transit, dissolution, and absorption of orally administered drugs (Tiny‐TIM)^13^ showed consistent performances for both batches. The form of the drug substance in both tablet batches was also shown to be the same and to match that of the drug substance at initial manufacture. Other work was conducted to understand the potential therapeutic window, integrating non‐clinical exposure‐response data with human safety and pharmacokinetics data.

These investigations led to the conclusion that there was a significantly narrowed predicted therapeutic window due to the combination of unexpectedly high and unexplained pharmacokinetic variability and exposure‐related AEs (headaches with or without associated symptoms) that were observed in the study. Therefore, the study was terminated.

## Conclusions

GSK286 was generally well tolerated by participants in the first part of this phase 1 study, but unexpectedly high pharmacokinetic variability and exposure‐related AEs in the second part led to termination of the study.

## Author Contributions

Emma Ilsley, Gareth Maher‐Edwards, Catherine Muya, Katie Rolfe, Raman Sharma, Simon Tiberi, and David Barros‐Aguirre contributed to the study concept or design. Edward Banham‐Hall was responsible for data acquisition. All authors performed data analysis and/or data interpretation. All authors contributed to the development of the manuscript by reviewing and providing input. All authors had full access to the data and gave final approval before submission. All authors agree to be accountable for all aspects of the work.

## Conflicts of Interest

All authors are GSK employees and hold financial equities in the company. Gareth Maher‐Edwards, Katie Rolfe, and David Barros‐Aguirre hold financial equities in Haleon. David Barros‐Aguirre also reports patents planned, issued or pending. All authors declare no other financial and non‐financial relationships and activities.

## Funding

GSK sponsored and funded the study (NCT04472897) and was involved in all aspects of the research, including data analysis. GSK also covered all costs related to the development and publication of this manuscript. This project, which led to the present publication, is part of the ERA4TB initiative and received support from the Innovative Medicines Initiative 2 Joint Undertaking (JU) under grant agreement no. 853989.

## Supporting information



Supporting information

## Data Availability

Please refer to GSK weblink to access GSK's data sharing policies and as applicable seek anonymized subject level data via the link https://www.gsk‐studyregister.com/en/.
