# Supplementary material for: Randomized, Placebo‐Controlled, First‐in‐Human Study of the Safety, Tolerability, and Pharmacokinetics of Single and Repeat Oral Doses of Novel Antitubercular Drug Candidate, GSK2556286, in Healthy Adults
Source: J Clin Pharmacol. 2026 May 15;66:e70203. doi: 10.1002/jcph.70203 (PMC13178715; doi:10.1002/jcph.70203)
Supplement: Supplementary file 1 — Supporting information [file JCPH-66-0-s001.docx]

# SUPPLEMENTARY MATERIAL

# Table S1. Plasma pharmacokinetic parameters of GSK2556286 in part A (single dose) and part B (repeated dose)

| **Parameter** | **Treatment, mg (cohort)** | **Day** | **Part A, single dose** | | |
| --- | --- | --- | --- | --- | --- |
|  |  |  | **N** | **Geometric mean** | **CV_b_ (%)** |
| AUC_0-t_ (h*ng/mL) | 25 (1A) | 1 | 6 | 191.74 | 191.60 |
|  | 75 (2A) |  | 6 | 451.43 | 79.44 |
|  | 75 (4A) |  | 6 | 605.56 | 83.91 |
|  | 75 fed (5A) |  | 6 | 1224.24 | 64.18 |
|  | 225 (3A) |  | 3 | 2481.12 | 98.26 |
|  | 225 (6A) |  | 6 | 3932.22 | 57.35 |
|  | 225 fed (7A) |  | 6 | 4243.07 | 86.96 |
|  | 450 fed (8A) |  | 6 | 10,295.44 | 47.82 |
|  | 650 fed (9A) |  | 6 | 11,818.04 | 56.52 |
|  | 1000 fed (10A) |  | 5 | 34,107.33 | 87.13 |
| AUC_0-∞_ (h*ng/mL) | 25 (1A) | 1 | 1 | 349.32 | - |
|  | 75 (2A) |  | 3 | 807.06 | 79.74 |
|  | 75 (4A) |  | 5 | 769.68 | 80.76 |
|  | 75 fed (5A) |  | 6 | 1306.09 | 63.96 |
|  | 225 (3A) |  | 3 | 2657.62 | 95.18 |
|  | 225 (6A) |  | 4 | 3938.72 | 47.94 |
|  | 225 fed (7A) |  | 6 | 4291.69 | 85.63 |
|  | 450 fed (8A) |  | 6 | 10,336.15 | 47.77 |
|  | 650 fed (9A) |  | 6 | 11,855.60 | 56.48 |
|  | 1000 fed (10A) |  | 4 | 43,153.67 | 69.00 |
| C_max_ (ng/mL) | 25 (1A) | 1 | 6 | 64.67 | 74.05 |
|  | 75 (2A) |  | 6 | 129.28 | 70.60 |
|  | 75 (4A) |  | 6 | 226.93 | 67.22 |
|  | 75 fed (5A) |  | 6 | 285.85 | 34.59 |
|  | 225 (3A) |  | 3 | 741.18 | 71.29 |
|  | 225 (6A) |  | 6 | 861.16 | 75.64 |
|  | 225 fed (7A) |  | 6 | 700.08 | 63.47 |
|  | 450 fed (8A) |  | 6 | 1870.43 | 35.13 |
|  | 650 fed (9A) |  | 6 | 1806.42 | 20.96 |
|  | 1000 fed (10A) |  | 5 | 3384.43 | 26.77 |
|  |  |  |  | **Median** | **Min/max** |
| T_1/2_ (h) | 25 (1A) | 1 | 0 | - | - |
|  | 75 (2A) |  | 1 | 1.96 | 2.0/2.0 |
|  | 75 (4A) |  | 4 | 1.70 | 1.4/2.8 |
|  | 75 fed (5A) |  | 6 | 1.68 | 1.4/5.1 |
|  | 225 (3A) |  | 2 | 3.74 | 1.6/5.9 |
|  | 225 (6A) |  | 4 | 2.89 | 2.5/6.3 |
|  | 225 fed (7A) |  | 6 | 3.71 | 1.8/6.1 |
|  | 450 fed (8A) |  | 6 | 4.29 | 1.9/5.2 |
|  | 650 fed (9A) |  | 6 | 4.51 | 1.7/8.3 |
|  | 1000 fed (10A) |  | 4 | 5.30 | 4.9/6.7 |
| T_max_ (h) | 25 (1A) | 1 | 6 | 1.51 | 0.8/3.0 |
|  | 75 (2A) |  | 6 | 1.75 | 0.8/3.0 |
|  | 75 (4A) |  | 6 | 0.77 | 0.5/4.0 |
|  | 75 fed (5A) |  | 6 | 3.00 | 1.5/4.0 |
|  | 225 (3A) |  | 3 | 0.75 | 0.5/2.0 |
|  | 225 (6A) |  | 6 | 1.75 | 0.5/4.0 |
|  | 225 fed (7A) |  | 6 | 4.00 | 1.1/4.0 |
|  | 450 fed (8A) |  | 6 | 3.00 | 0.3/6.0 |
|  | 650 fed (9A) |  | 6 | 3.50 | 1.5/6.0 |
|  | 1000 fed (10A) |  | 5 | 4.00 | 1.5/6.0 |
|  |  |  | **Part B, repeat dose** | | |
|  |  | **Day** | **N** | **Geometric mean** | **CV_b_ (%)** |
| AUC_0-t_ (h*ng/mL) | 225 (1B) | 1 | 7 | 3583.83 | 65.23 |
|  |  | 9 | 6 | 4334.73 | 54.52 |
|  |  | 11 | 6 | 4813.76 | 43.51 |
|  |  | 14 | 6 | 4099.12 | 32.21 |
|  | 650 (2B) | 1 | 6 | 30,603.63 | 90.49 |
|  |  | 14 | 1 | 125,159.44 | - |
| AUC_0-∞_ (h*ng/mL) | 225 (1B) | 1 | 7 | 3635.21 | 63.89 |
|  |  | 9 | 6 | 4582.71 | 53.59 |
|  |  | 11 | 6 | 4848.86 | 43.59 |
|  |  | 14 | 6 | 4139.08 | 32.55 |
|  | 650 (2B) | 1 | 5 | 25,823.99 | 77.03 |
|  |  | 14 | 0 | - | - |
| AUC_0-τ_ (h*ng/mL) | 225 (1B) | 1 | 7 | 3602.53 | 65.34 |
|  |  | 9 | 6 | 4340.18 | 54.47 |
|  |  | 11 | 6 | 4835.24 | 43.26 |
|  |  | 14 | 6 | 4100.79 | 32.14 |
|  | 650 (2B) | 1 | 6 | 30,690.45 | 90.62 |
|  |  | 14 | 1 | 125,159.44 | - |
| C_max_ (ng/mL) | 225 (1B) | 1 | 7 | 978.51 | 64.36 |
|  |  | 9 | 6 | 1038.47 | 66.92 |
|  |  | 11 | 6 | 1299.04 | 29.62 |
|  |  | 14 | 6 | 899.55 | 33.82 |
|  | 650 (2B) | 1 | 6 | 4016.33 | 43.45 |
|  |  | 14 | 1 | 10,100.00 | - |
| C_τ_ (ng/mL) | 225 (1B) | 1 | 7 | 2.94 | 175.20 |
|  |  | 2 | 7 | 3.07 | 210.34 |
|  |  | 3 | 7 | 4.81 | 147.43 |
|  |  | 6 | 6 | 2.79 | 128.84 |
|  |  | 7 | 6 | 2.24 | 135.16 |
|  |  | 8 | 6 | 2.80 | 177.12 |
|  |  | 9 | 6 | 14.06 | 123.11 |
|  |  | 11 | 5 | 3.10 | 178.61 |
|  |  | 14 | 6 | 4.94 | 145.56 |
|  | 650 (2B) | 1 | 6 | 166.68 | 517.17 |
|  |  | 2 | 6 | 163.44 | 2601.66 |
|  |  | 3 | 4 | 187.85 | 1759.00 |
|  |  | 6 | 2 | 33.91 | >9999 |
|  |  | 7 | 1 | 1400.00 | - |
|  |  | 8 | 0 | - | - |
|  |  | 14 | 1 | 2480.00 | - |
|  |  |  |  | **Median** | **Min/max** |
| T_1/2_ (h) | 225 (1B) | 1 | 6 | 2.76 | 1.6/4.0 |
|  |  | 9 | 5 | 5.08 | 2.3/6.5 |
|  |  | 11 | 6 | 2.36 | 1.8/3.7 |
|  |  | 14 | 5 | 3.27 | 1.9/3.7 |
|  | 650 (2B) | 1 | 5 | 4.35 | 3.0/6.0 |
|  |  | 14 | 0 | - | - |
| T_max_ (h) | 225 (1B) | 1 | 7 | 2.00 | 0.5/4.0 |
|  |  | 9 | 6 | 0.63 | 0.5/2.0 |
|  |  | 11 | 6 | 1.50 | 0.5/3.0 |
|  |  | 14 | 6 | 3.00 | 0.8/4.0 |
|  | 650 (2B) | 1 | 6 | 3.50 | 1.5/4.0 |
|  |  | 14 | 1 | 4.00 | 4.0/4.0 |

AUC_0-t_, area under the concentration-time curve from time 0 to the time of the last quantifiable concentration; AUC_0-∞_, area under the concentration-time curve from time 0 to infinity; AUC_0-τ_, area under the concentration-time curve from time 0 to the end of dosing period; C_max_, maximum observed concentration; C_τ_, concentration at end of dosing interval; CV_b_, between-participant coefficient of variation; Max, maximum; Min, minimum; N, number of participants; T_1/2_, terminal phase half-life; T_max_, time to reach maximum observed plasma concentration.

# Table S2. Effect of food on log-transformed plasma pharmacokinetic parameters of GSK2556286 in part A (single dose)

|  |  | **Condition: Fed** | | **Condition: Fasted** | | **Fed:fasted ratio (90% CI)** |
| --- | --- | --- | --- | --- | --- | --- |
| **Parameter** | **Treatment (mg)** | **N** | **Geometric LS mean** | **N** | **Geometric LS mean** |  |
| AUC_0-t_ (h*ng/mL) | 75 | 6 | 1224.24 | 6 | 605.56 | 2.02 (1.05, 3.89) |
|  | 225 | 6 | 4243.07 | 6 | 3932.22 | 1.08 (0.56, 2.08) |
| AUC_0-∞_ (h*ng/mL) | 75 | 6 | 1306.09 | 5 | 769.68 | 1.70 (0.86, 3.35) |
|  | 225 | 6 | 4291.69 | 4 | 3938.72 | 1.09 (0.53, 2.25) |
| C_max_ (ng/mL) | 75 | 6 | 285.85 | 6 | 226.93 | 1.26 (0.72, 2.21) |
|  | 225 | 6 | 700.09 | 6 | 861.16 | 0.81 (0.46, 1.43) |
| T_1/2_ (h) | 75 | 6 | 2.04 | 4 | 1.83 | 1.12 (0.66, 1.88) |
|  | 225 | 6 | 3.44 | 4 | 3.39 | 1.02 (0.60, 1.71) |
|  |  |  | **Median** |  | **Median** | **Difference (90% CI)** |
| T_max_ (h) | 75 | 6 | 3.00 | 6 | 0.77 | 1.50 (0.00, 2.48) |
|  | 225 | 6 | 4.00 | 6 | 1.75 | 1.75 (0.00, 3.23) |

AUC_0-t_, area under the concentration-time curve from time 0 to the time of the last quantifiable concentration; AUC_0-∞_, area under the concentration-time curve from time 0 to infinity; CI, confidence interval; C_max_, maximum observed concentration; LS, least squares; N, number of participants; T_1/2_, terminal phase half-life; T_max_, time to reach maximum observed plasma concentration.

# Table S3. Effect of food on log-transformed plasma pharmacokinetic parameters of GSK2556286 in part B (repeat dose)

| **Parameter** | **Treatment (mg)** | **Condition: Fed (HF or Std meal)** | | **Condition: Fasted** | | **Fed:fasted ratio (90% CI)** |
| --- | --- | --- | --- | --- | --- | --- |
|  |  | **N** | **Geometric LS mean** | **N** | **Geometric LS mean** |  |
| AUC_0-t_ (h*ng/mL) | 225 | 6 | 4813.76 (HF) | 6 | 4334.73 | 1.11 (0.96, 1.29) |
|  | 225 | 6 | 4099.12 (Std) | 6 | 4334.73 | 0.95 (0.82, 1.10) |
| AUC_0-∞_ (h*ng/mL) | 225 | 6 | 4848.86 (HF) | 6 | 4582.71 | 1.06 (0.90, 1.25) |
|  | 225 | 6 | 4139.08 (Std) | 6 | 4582.71 | 0.90 (0.77, 1.06) |
| AUC_0-τ_ (h*ng/mL) | 225 | 6 | 4835.24 (HF) | 6 | 4340.18 | 1.11 (0.96, 1.29) |
|  | 225 | 6 | 4100.79 (Std) | 6 | 4340.18 | 0.95 (0.82, 1.09) |
| C_max_ (ng/mL) | 225 | 6 | 1299.05 (HF) | 6 | 1038.47 | 1.25 (0.81, 1.94) |
|  | 225 | 6 | 899.56 (Std) | 6 | 1038.47 | 0.87 (0.56, 1.34) |
| C_τ_ (ng/mL) | 225 | 5 | 3.49 (HF) | 6 | 14.07 | 0.25 (0.14, 0.45) |
|  | 225 | 6 | 4.94 (Std) | 6 | 14.07 | 0.35 (0.20, 0.61) |
| T_1/2_ (h) | 225 | 6 | 2.50 (HF) | 5 | 4.57 | 0.55 (0.39, 0.77) |
|  | 225 | 5 | 3.03 (Std) | 5 | 4.57 | 0.66 (0.47, 0.94) |
|  |  |  | **Median** |  | **Median** | **Difference (90% CI)** |
| T_max_ (h) | 225 | 6 | 1.50 (HF) | 6 | 0.63 | 0.75 (-0.63, 2.50) |
|  | 225 | 6 | 3.00 (Std) | 6 | 0.63 | 1.88 (0.27, 3.00) |

AUC_0-t_, area under the concentration-time curve from time 0 to the time of the last quantifiable concentration; AUC_0-∞_, area under the concentration-time curve from time 0 to infinity; AUC_0-τ_, area under the concentration-time curve from time 0 to the end of dosing period; CI, confidence interval; C_max_, maximum observed concentration; C_τ_, concentration at end of dosing interval; HF, high fat; LS, least squares; N, number of participants; Std, standard; T_1/2_, terminal phase half-life; T_max_, time to reach maximum observed plasma concentration.
